# hPL promotes osteogenic differentiation of stem cells in 3D scaffolds

**DOI:** 10.1371/journal.pone.0215667

**Published:** 2019-05-07

**Authors:** Hanan Jafar, Duaa Abuarqoub, Nidaa Ababneh, Maram Hasan, Shrouq Al-Sotari, Nazneen Aslam, Mohammed Kailani, Mohammed Ammoush, Ziad Shraideh, Abdalla Awidi

**Affiliations:** 1 Cell Therapy Center, The University of Jordan, Amman, Jordan; 2 School of Medicine, The University of Jordan, Amman, Jordan; 3 Department of Chemistry, School of Sciences, The University of Jordan, Amman, Jordan; 4 Dental Department, King Hussein Medical Center (KHMC), Royal Medical Service, Amman, Jordan; 5 Department of Biological Sciences, School of Sciences, The University of Jordan, Amman, Jordan; Northeastern University, UNITED STATES

## Abstract

Human platelet lysate (hPL) has been considered as the preferred supplement for the xeno-free stem cell culture for many years. However, the biological effect of hPL on the proliferation and differentiation of dental stem cells combined with the use of medical grade synthetic biomaterial is still under investigation. Thus, the optimal scaffold composition, cell type and specific growth conditions, yet need to be formulated. In this study, we aimed to investigate the regenerative potential of dental stem cells seeded on synthetic scaffolds and maintained in osteogenic media supplemented with either hPL or xeno-derived fetal bovine serum (FBS). Two types of dental stem cells were isolated from human impacted third molars and intact teeth; stem cells of apical papilla (SCAP) and periodontal ligament stem cells (PDLSCs). Cells were expanded in cell culture media supplemented with either hPL or FBS. Consequently, proliferative capacity, immunophenotypic characteristics and multilineage differentiation potential of the derived cells were evaluated on monolayer culture (2D) and on synthetic scaffolds fabricated from poly ’lactic-co-glycolic’ acid (PLGA) (3D). The functionality of the induced cells was examined by measuring the concentration of osteogenic markers ALP, OCN and OPN at different time points. Our results indicate that the isolated dental stem cells showed similar mesenchymal characteristics when cultured on hPL or FBS-containing culture media. Scanning electron microscopy (SEM) and H&E staining revealed the proper adherence of the derived cells on the 3D scaffold cultures. Moreover, the increase in the concentration of osteogenic markers proved that hPL was able to produce functional osteoblasts in both culture conditions (2D and 3D), in a way similar to FBS culture. These results reveal that hPL provides a suitable substitute to the animal-derived serum, for the growth and functionality of both SCAP and PDLSCs. Thus the use of hPL, in combination with PLGA scaffolds, can be useful in future clinical trials for dental regeneration.

## Introduction

The term periodontium refers to the combination of dental tissues that support the teeth and they are developmentally, topographically, and functionally related [1]. Periodontitis-associated tissue loss is the most common cause of tooth loss among adult population in the developing countries [2]. Periodontitis is an infectious and inflammatory disease of the supportive tissues of the teeth, which comprises of gingival, cementum, alveolar bone and periodontal ligament (PDL)[3]. PDL is the connective tissue fiber that runs between alveolar bone and cementum. As the periodontal disease progresses, it degenerates the connective tissue fibers on the periodontal ligament (PDL) along with other tissues, leading to tooth loss. The high prevalence of the periodontal disease and the critical role of the PDL in maintaining the physiological function of the tooth have increased the focus of current research on PDL tissue engineering. Due to the limited regenerative ability of PDL, regeneration of the periodontal apparatus composed of bone, PDL and cementum remains a challenge. Hence, a complete regeneration of the periodontium is still unattainable [4, 5].

Stem cell therapy represents a promising new approach for the regeneration of defective tissues or functions through the transplantation of cells that have the potential to specifically repair the degenerated tissues. Mesenchymal stem cells (MSCs) hold a great promise in regenerative medicine, due to their multipotency and tissue specificity [6]. Recently, dental tissues-derived MSCs have gained considerable attention as an attractive source for maxillofacial regenerative therapy. To date, eight unique populations of dental tissue-derived MSCs have been isolated and characterized. Postnatal dental pulp stem cells (DPSCs) were the first human dental MSCs to be identified from pulp tissue [7]. Other dental MSC-like populations, such as stem cells from human exfoliated deciduous teeth (SHED) [8] http://onlinelibrary.wiley.com/doi/10.1002/stem.1909/full, periodontal ligament stem cells (PDLSCs) [9], dental follicle progenitor cells (DFPCs) [10], alveolar bone-derived MSCs (ABMSCs) [11], stem cells from apical papilla (SCAP) [12], tooth germ progenitor cells (TGPCs) [13], periapical cyst mesenchymal stem cells (hPCy-MSCs)[14] and gingival MSCs (GMSCs) have also been reported [15, 16]. Dental stem cells (DSCs) can differentiate into odontoblasts, adipocytes, neuronal-like cells, glial cells, osteoblasts, chondrocytes, melanocytes, myotubes and endothelial cells [7, 17–19].

Dental stem cells can be used for dental tissue regenerative therapy in terms of dentine formation, pulp revascularization and periodontal regeneration. SCAP can be easily isolated from human third molars and have shown a two- to three-fold higher proliferation rate than DPSCs and they are more committed to osteo/dentinogenicity. It has also been reported that transplantation of SCAPs into immunocompromised mice in a carrier matrix has resulted in the formation of a typical dentin-pulp-like structure, due to the presence of odontoblast-like cells [20]. Seo *et al*. identified stem cells in human PDL and found that PDLSCs implanted into nude mice generated cementum/PDL-like structures that resemble the native PDL. Taken together, these cells present new opportunities for dental tissue engineering and cellular therapy, particularly in dental tissue loss [9, 12, 21, 22].

In order to create human tissue products, several polymers have been tested for scaffold construction, to enhance the growth [12, 23–25] and differentiation of different cell types [26–28]. The copolymer poly lactic-co-glycolic acid (PLGA) has been considered as the most attractive polymeric candidate in a variety of biomedical applications, due to its biocompatibility, biodegradability, and the Food and Drug Administration (FDA) approval for human use [24, 29].

Currently, there is an active research going in the field of regenerative medicine for the optimization of scaffold construction and stem cell cultivation in the presence of stimulating growth factors. The multipotent differentiation properties of PDLSC were demonstrated by constructing multilayered cell sheets supported by woven polyglycolic acid [30]. Transplanted cell-seeded polyglycolic acid sheets have regenerated new bone, cementum, and well-oriented collagen fibers when inserted into root surfaces [31]. Current researches are focused on finding a way to develop an appropriate delivery system for engineering an efficient cell-based therapeutic tool for periodontal tissue regeneration. Flores *et al*. have shown that fibrin gels carrying several layers of PDL cells can be used as a delivery system for these cells [32]. Moreover, hydrogel scaffolds derived from decellularized bone extracellular matrix (bECM) showed an impact on the upregulation of the osteo-specific markers compared to other scaffolds[33]. Collagen sponge scaffolds seeded with PDL cells were successfully used for the regeneration of periodontal fenestration defects in beagle dogs [34].Additionally, the concept of the multipotency of scaffolds has highlighted the importance of the environment in directing cell homing and differentiation, when implants of Tibialis Anterior/tibial bone and masseter muscle/mandible bone were implanted in a murine model, resulted in finding muscle cells near the host muscle and by bone-cartilaginous tissues near the host bone[35]. Another delivery system for PDLSC based on a combination of bovine bone with human dentin was shown to be effective because these stem cells formed a cementum-like complex in subcutaneous dorsum pockets of immunocompromised mice [36].

Traditionally, cells are expanded in media supplemented with animal serum such as FBS. However, due to the issues of disease transmission and antigenicity, focus has been shifted to the use of xeno–free products for safer and more reproducible cell culture conditions. Additionally, it is becoming crucial to apply the good manufacturing practice (GMP) to cellular products intended for therapeutic use. Recently, hPL has drawn a lot of attention as a substitute of FBS for clinical-grade cell expansion [37–41]. It has been shown previously that platelet granules are rich in many growth factors such as: platelet derived growth factor (PDGF), fibroblast growth factor (FGF), insulin growth factor (IGF), and transforming growth factor-b (TGFB) [42, 43].

Many studies have reported the effectiveness of using hPL in clinical trials for both autologous and allogeneic transplantations [44,45]. Intra-articular autologous hPL injection in early and intermediate knee osteoarthritis significantly improved the symptoms of pain and stiffness [45].

In this study, we aimed to investigate the effect of hPL on the proliferation capacity and particularly the osteogenic differentiation potential of dental stem cells cultured in monolayer (2D) and PLGA scaffolds (3D).

## Materials and methods

### Platelet lysate preparation

The study protocol was approved by the Institutional Review Board (IRB) at the Cell Therapy Center (CTC) / University of Jordan (99/2014/TRBJ). All subjects have signed informed consents in accordance with Helsinki declaration prior to sample collection. hPL samples were prepared as described previously [46]. Briefly, platelet bags from several donors were pooled and centrifuged for 10 min at 110 xg to prepare platelet rich pellet [43, 47, 48]. The supernatant which composed of platelet poor plasma (PPP) was discarded, and the pellet was resuspended in the remaining volume to produce platelet rich plasma (PRP). Then, PRP was centrifuged for 10 min at 3060 xg and adjusted to 1×10^6^ cells/μl. After that, PRP was activated by three subsequent freeze-thaw cycles, at −80°C and 37°C, respectively and then the resulting hPL was centrifuged at 3060 xg for 10 min. After that, hPL was filtered using 0.22μm filters before being used in cell culture media. hPL samples from at least five donors were pooled to reduce donor variability [47].

### Isolation and expansion of dental stem cells (SCAP and PDLSCs)

For the isolation of SCAPs, impacted third molar samples were collected from three different donors as the following: 18, 19 and 25 year-old patients. Similarly, for the isolation of PDLSCs, intact teeth were extracted from three random donors. All teeth were primarily extracted for either orthodontic or surgical reasons in a clinical setting. All donors have signed an informed consent prior to sample collection. Teeth were submerged in transport media, consisting of alpha-modification of Eagle’s Medium (α-MEM, Gibco, Life Technologies, USA), supplemented with 1% penicillin/streptomycin and amphotericin B (Invitrogen, Life Technologies, USA) and stored at 4°C until further use.

SCAP and PDLSCs were isolated as described previously using enzymatic digestion method [12, 49]. First, teeth were disinfected with 1X PBS for three times. For SCAP isolation, the apical papilla was cut away from the immature roots using a sterile scalpel and blade. For PDLSCs, the PDL tissue was removed from mid third level of root. On both procedures, tissues were digested in a solution containing 3mg/ml collagenase type I (GIBCO, Germany) and 4mg/ml dispase (GIBCO, Germany) for 1h at 37** °**C. Single-cell suspensions were obtained by passing the cells through 70 μm strainer (BD Biosciences, Germany). Cells were centrifuged at 1000 rpm for 5 minutes and pellets were re-suspended in 1 ml of αMEM culture medium supplemented with 100 mM L-ascorbic acid phosphate (Sigma-Aldrich, USA), 100 mg/ml streptomycin (Invitrogen, USA), 2mM L-glutamine (Invitrogen, USA), 0.25 mg/ml Amphotericin B (Invitrogen, USA) and 100 units/ml penicillin (Invitrogen, USA)]. In monolayer culture, primary cells from single cell suspensions were seeded at a density of 10,000 cell/cm^2^ in 6 well plates and maintained in the same cell culture media described above, with either 5% hPL or 10% FBS, while serum free medium (SFM) was kept as a negative control in all performed experiments. The cells were incubated in a humidified incubator at 37**°**C and 5% CO_2_. The culture media was exchanged every three days until the cells reached 70–80% confluence, then cells were harvested using 0.25% trypsin/EDTA (Gibco, Life Technologies,USA). Cells at passage 3 through 5 were utilized for further experimentation, and all experiments were performed in triplicate.

### Characterization of SCAP and PDLSCs in monolayer culture

#### Immunophenotyping by flow cytometry

For the identification of SCAP and PDLSCs cell surface markers, cells at passage 3 cultured in media supplemented with either 10% FBS or 5% hPL were trypsinized and washed twice with PBS. Following that, 1×10^6^ cells/ml from each type were incubated with different fluorescinated antibodies using Stem flow kit (BD Biosciences, USA). Antibody concentration and the staining procedure were based on the manufacturer’s instructions (BD, USA). The stem cell expression markers were analysed by FACS DIVA 7 software using Flourescien activated cell sorter FACS Canto II (BD, USA).

#### Growth evaluation

To determine the effect of using different serum types on the proliferative capacity of the isolated SCAP and PDLSCs, cells were evaluated using two different assays: MTT (3-(4,5-Dimethylthiazol-2-yl)-2,5-Diphenyltetrazolium Bromide) and Colony-forming efficiency assay (CFE).

To evaluate the effect of the serum on cell proliferation rate, 5000 cells of either SCAP or PDLSCs were seeded into 96 well plates and maintained in culture media consisting of either 5% hPL or 10% FBS in addition to SFM for 3 days. Briefly, 20 μl of 5mg/ml MTT reagent were added to each well, and incubated for 4 hours at 37°C and 5% CO_2_. Next, the media were aspirated and 100 μl of DMSO were added and incubated for further 15 minutes. The proliferation rate was determined after 24, 48 and 72 hours. Optical density was measured at 570 nm using a microplate reader (GloMax-multi microplate reader, Promega, USA).

CFE was also performed to assess the self-renewal capacity of the isolated cells from passage 1, 2 and 3. Briefly, 500 cells were seeded in 20 mm dish and maintained under the same culture media mentioned above. The media were changed every three days, and after 2 weeks cells were stained with crystal violet dye to identify the number of colonies produced. Colonies containing 50 cells or more were counted, and CFE was determined as percentages.

#### Multilineage differentiation

For multilineage differentiation, the protocol was carried out as previously described [29]. SCAP and PDLSCs were plated at a seeding density of 1×10^5^ cells/well in 6-well plates, and maintained in growth media containing either 10% FBS or 5% hPL until they reached 70% confluence and then differentiation media were added accordingly. For adipogenic differentiation, cells were incubated in adipogenic medium for 3 weeks and medium was exchanged every other day. Induced cells with oil droplets formation were stained with oil red O dye (Sigma, USA). Adipogenic medium was composed of α-MEM supplemented with 1 mM dexamethasone (Sigma, USA), 1 mg/mL insulin (Sigma, USA), 0.5mM 3-isobutyl-1-methylxanthine (Sigma, USA), and either 10% FBS or 5% hPL.

 For osteogenic differentiation, cells were incubated for 14 days in differentiation media composed of α-MEM supplemented with 10 mM β-glycerophosphate (Sigma, USA), 50 μg/mL L-ascorbic acid 2-phosphate (Sigma, USA), 1 μM dexamethasone (Sigma, USA), and either 10% FBS or 5% hPL, and differentiated cells were stained with alizarin red stain (Sigma, USA) for calcium deposition.

### Characterization of SCAP and PDLSCs on PLGA scaffolds (3D)

#### PLGA scaffold preparation

Poly-lactic co-glycolic acid (PLGA) scaffolds with a lactic acid: glycolic acid ratio of 75:25 was prepared as previously described [50, 51]. Briefly, sugar particles measuring 160–200 μm were dispersed in PLGA solution in chloroform. The polymer was precipitated and sugar crystals were leached out in water and then scaffolds were fabricated to measure 1X mm^3^. Characterization of the prepared PLGA scaffolds was determined by using the Fourier Transform Infrared (FTIR) Spectroscopy (Madison,WI). The Fourier transform infrared (FTIR) analysis was conducted as neat films over NaCl plates on Thermo Nicolet 670 Nexus FTIR spectrophotometer after averaging 48 scans with a resolution of 4 cm^−1^.

The general characteristics of PLGA polymer and its raw material were assessed by determining the thermal stability using Thermogravimetric Analyzer (TGA/DSC2) (Mettler-Toledo, Switzerland). Samples were heated to 400 °C at a rate of 10 °C/min under a dry nitrogen atmosphere purging at a flow rate of 30 mL/min. After that, glass transition temperature (Tg) was examined by Differential Scanning Calorimetry (DSC) (Shimadzu 60A plus, Japan). For the (Tg) the measurements were carried out under a dry nitrogen atmosphere at a flow of 40 mL/min. The samples were subjected to cooling and heating cycles from -50°C to 100°C with heating and cooling rates of 10°C/min.

#### Scanning electron microscopy (SEM)

For SEM, scaffolds seeded with cells were fixed using 2.5% gluteraldehyde in 0.1M cacodylate buffer for 1 hour at 37°C, then washed three times with 0.2M cacodylate buffer and finally fixed in 1% osmium tetraoxide for 1 hour. Some scaffolds were dried using a critical point dryer and then surface coated with gold, while others were kept immersed in distilled water. The dried scaffolds were imaged at high vacuum and 10kV acceleration voltage (Versa 3D, FEI). The wet scaffolds were imaged using SEM mode at 100% humidity. The liquid was slowly dehydrated from the surfaces of these scaffolds by decreasing the pressure gradually inside the chamber and therefore maintaining the three-dimensional structure of the scaffolds [37].

#### Seeding and characterization of SCAP and PDLSCs on PLGA scaffolds

For seeding DSCs on scaffolds, scaffolds were sterilized using 70% alcohol for 2 hours and then coated with 10 ng/ul fibronectin (Sigma, USA) and incubated overnight at 4°C. After that, PLGA scaffolds were distributed into 24-well plate and then SCAP and PDLSCs were seeded individually on scaffolds at seeding density of 5x10^5^ cells/scaffold and incubated for 2 hours at 37°C and 5% CO_2_. Finally, cell culture media with either 5% hPL or 10% FBS were added to scaffolds and maintained under the same conditions.

To evaluate the migration efficiency of the cultured cells, seeded scaffolds were fixed in 3.7% formaldehyde, then dehydrated in a graded ethanol series of 90, 75 and 50% for 10 min each, respectively. The scaffolds were embedded in paraffin wax and hardened on a cold block overnight. Sections measuring 10 μm were obtained using a rotary microtome (Thermo Scientific, USA). Sections were then mounted on glass slides and stained with hematoxylin and eosin as described previously [29].

### Quantitative evaluation of Osteogenesis in 2D and 3D cultures

After osteogenic differentiation for 2D and 3D cultures, samples were collected at day 1, 7 and 14 of induction. The functionality of the cultured osteoblasts was examined by measuring certain osteogenic markers such as alkaline phosphatases [38], osteocalcin (OCN) and osteopontin (OPN) for cells seeded on PLGA scaffolds, compared to PLGA scaffolds cultured in cell culture media without the osteogenic supplements (blank control). The expression markers were assessed using the following human ELISA kits: Alkaline phosphatase assay kit (Abcam, USA), Human osteocalcin Elisa kit OCN (R&D, USA) and Human osteopontin Elisa Kit OPN (R&D, USA), according to the manufacturer’s instructions. The absorbance was measured at 405 nm for ALP and 450 nm for OCN and OPN, using a microplate reader (GloMax-multi microplate reader, Promega, USA).

### Statistical analysis

All experiments were performed in triplicates and samples were collected from three independent runs. Data were analyzed using Microsoft Excel and GraphPad Prism software. Quantitative data were represented as mean ± standard deviation. Data were evaluated by paired Student’s t-test. ANOVA test followed by Bonferroni post-test was used to analyze differences between three or more experimental points (P<0.05).

## Results

### Characterization of SCAP and PDLSCs on monolayer culture

**Fig 1:**
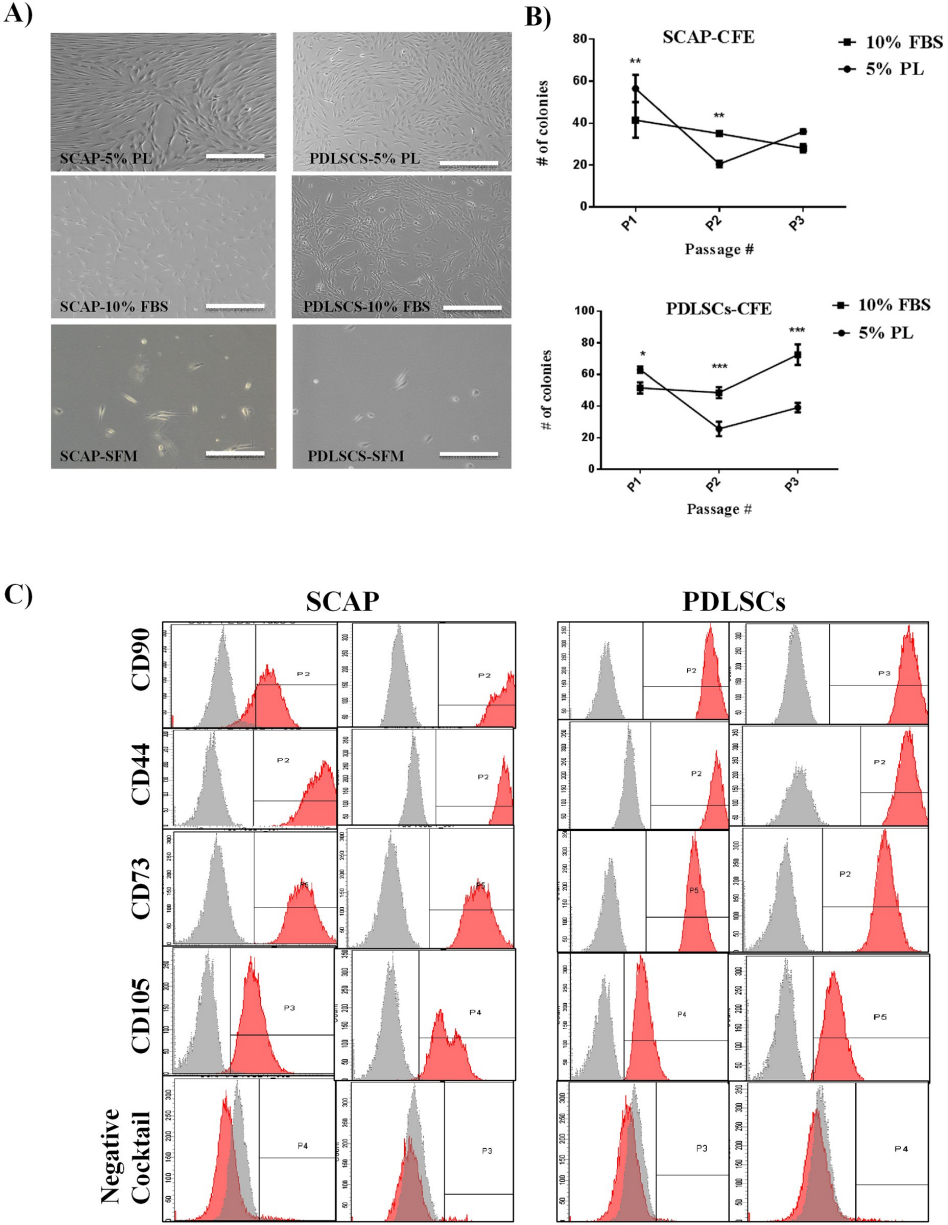
Characterization of SCAP and PDLSCs. A) Morphological appearance of SCAP and PDLSCs at P3, cultured under PL, FBS and SFM. Both cell types showed MSC appearance, spindle to elongated neuronal and fibroblastic-like morphology, under all sera used (scale bar = 100μm). **B)** Colony formation assay of SCAP and PDLSCs cultured in different serum types either 5% PL or 10% FBS. Colony formation was visualized by crystal violet staining. A statistical significant difference was observed between cells cultured in 10% FBS and PL (P<0.05). **C)** Flow cytometric analysis of SCAP and PDLSC cell surface expression markers. Histograms show that cells are positive for the following MSCs markers: CD90, CD44, CD73, and CD105), and negative expression of the negative cocktail markers including CD45, CD34, and HLA-DR.

The cell growth and morphological characteristics of the isolated SCAP and PDLSCs and maintained on three different serum types were assessed under the inverted phase-contract microscope. Both cell types showed MSC appearance, spindle to elongated cell body shape, neuronal and fibroblastic-like morphology under all sera used (Fig 1A).

**Fig 2:**
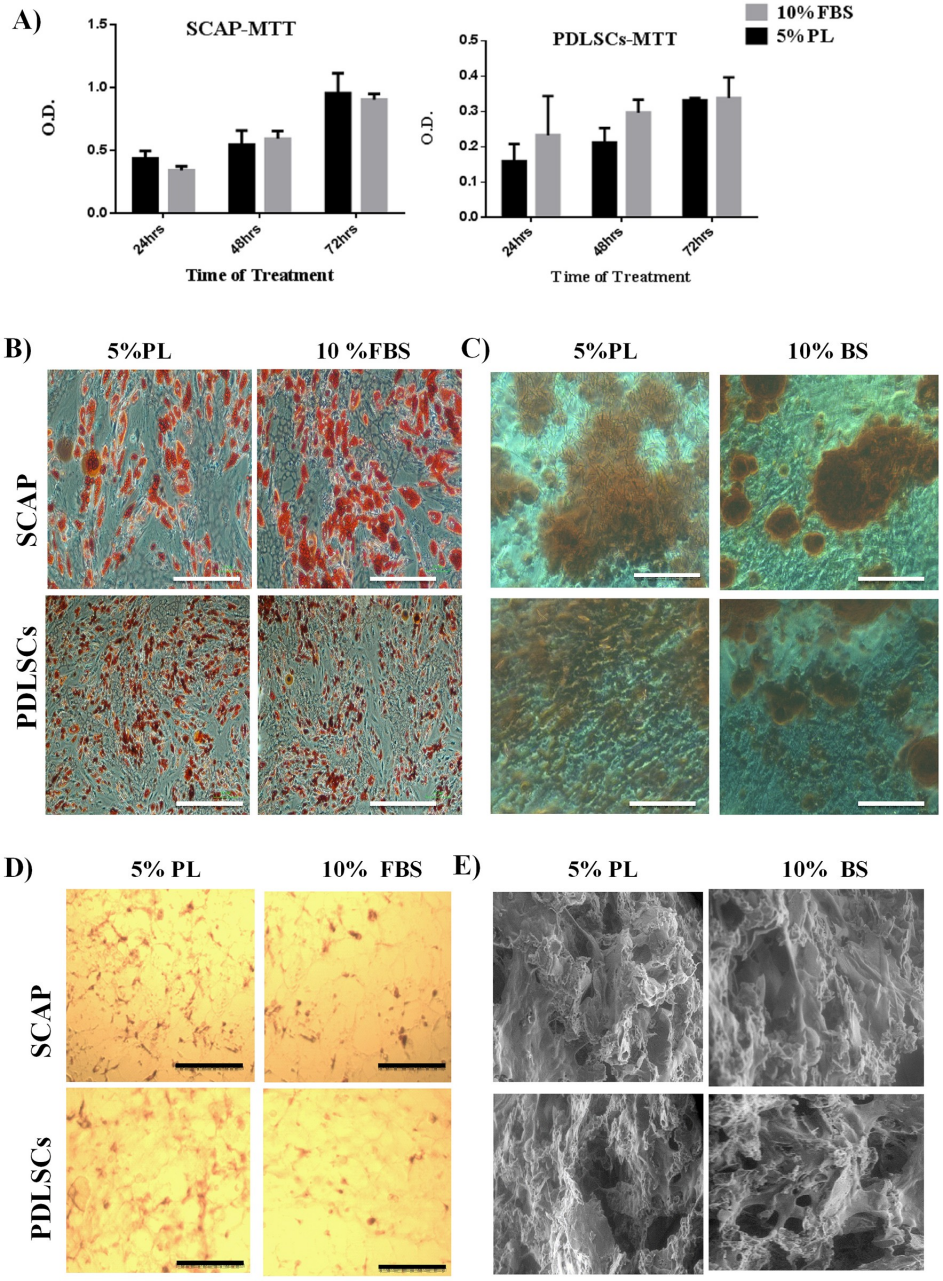
Evaluation of different serum types on the proliferation, differentiation and growth capacity of SCAP and PDLSCs. A) Proliferation was measured using MTT assay after 72 hours. Statistical difference was observed between SFM and the two serum types (P<0.05). No statistical difference was observed in the proliferation capacity of both cell types cultured either on FBS or PL. B&C) Multineage differentiation of SCAP and PDLSCs, induced by Adipogenic differentiation (Scale Bar = 10μm), and Osteogenic differentiation (Scale Bar = 100μm) using induction media supplemented with either 5%PL or 10%FBS, respectively. D) Histological analysis of cells seeded on PLGA scaffold, H&E staining showing high degree of cell infiltration. (Scale Bar = 2μm). E) SEM images of cells seeded on PLGA scaffold showing highly extended morphology within the pores of the scaffold. (Scale bar = 100μm).

For CFE, cells cultured in 10% FBS have shown a statistically significant increase in their colony forming efficiency when compared to cells cultured in 5% hPL (P<0.05). Whereas, both PDLSCs and SCAP cultured in SFM did not form any colonies as shown in (Fig 2B) (S1 Dataset). Cultures expanded in media supplemented with 10% FBS showed a significantly higher value when compared to their counterparts expanded in 5% hPL (P<0.05).

The isolated populations from SCAP and PDLSCs showed positive expression of MSC markers (CD90, CD44, CD73, and CD105) and negative for hematopoietic and endothelial cell markers (CD45, CD34 and HLA-DR) (Fig 1C). This was observed in all cultures regardless of the type of cells and serum used. This would indicate that the isolated populations showed MSC characteristics.

The evaluation of growth and proliferation of the isolated cultured cells was carried out by performing MTT proliferation assay. Regardless of the cell type, no statistical differences were observed in the proliferation of cells cultured on either hPL or FBS (Fig 2A) (S2 Dataset). However, a statistically significant difference was observed when the results of these cultures were compared to the SFM cultures (Fig 2A). SCAP and PDLSCs were successfully induced for adipogenesis and Osteo/odontogenesis lineages irrespective of the serum choice. (Fig 2B and 2C).

### Characterization of SCAP and PDLSCs on PLGA scaffolds

PLGA scaffold and its raw material were characterized with FTIR, DSC and TGA [50, 51]. For SCAP and PDLSCs the Infrared Red (IR) spectra were very similar (S1 Fig). Additionally, their DSC thermograms indicate that the glass transition temperature (Tg) value of the two samples was identical (S2 Fig). However, thermal stability of PLGA scaffold was higher compared to the raw material. This could be explained due to the large surface area of the scaffold (S3 Fig). Furthermore, the two PLGA samples have yielded a char residue, which indicates that the polymer is completely decomposed into gaseous products.

To visualize the topography and porosity of the scaffolds, PLGA scaffolds were imaged using SEM. The scaffolds showed high degree of porosity and pore size was variable, ranged between more than 50 μm to extremely fine ones of less than 5 μm diameter.

Scaffolds seeded with SCAP and PDLSCs were also processed and stained with H&E stain. Cells showed an even distribution within the pores of the scaffolds with a complex inter-connectivity pattern (Fig 2D). The topography of cellular adherence on PLGA scaffolds was also assessed using SEM. Several cells were observed to extend on the outer surface of the scaffolds and within their pores (Fig 2E).

### Comparison of osteo/odontogenesis differentiation potential of SCAP and PDLSCs in 2D and 3D cultures

**Fig 3:**
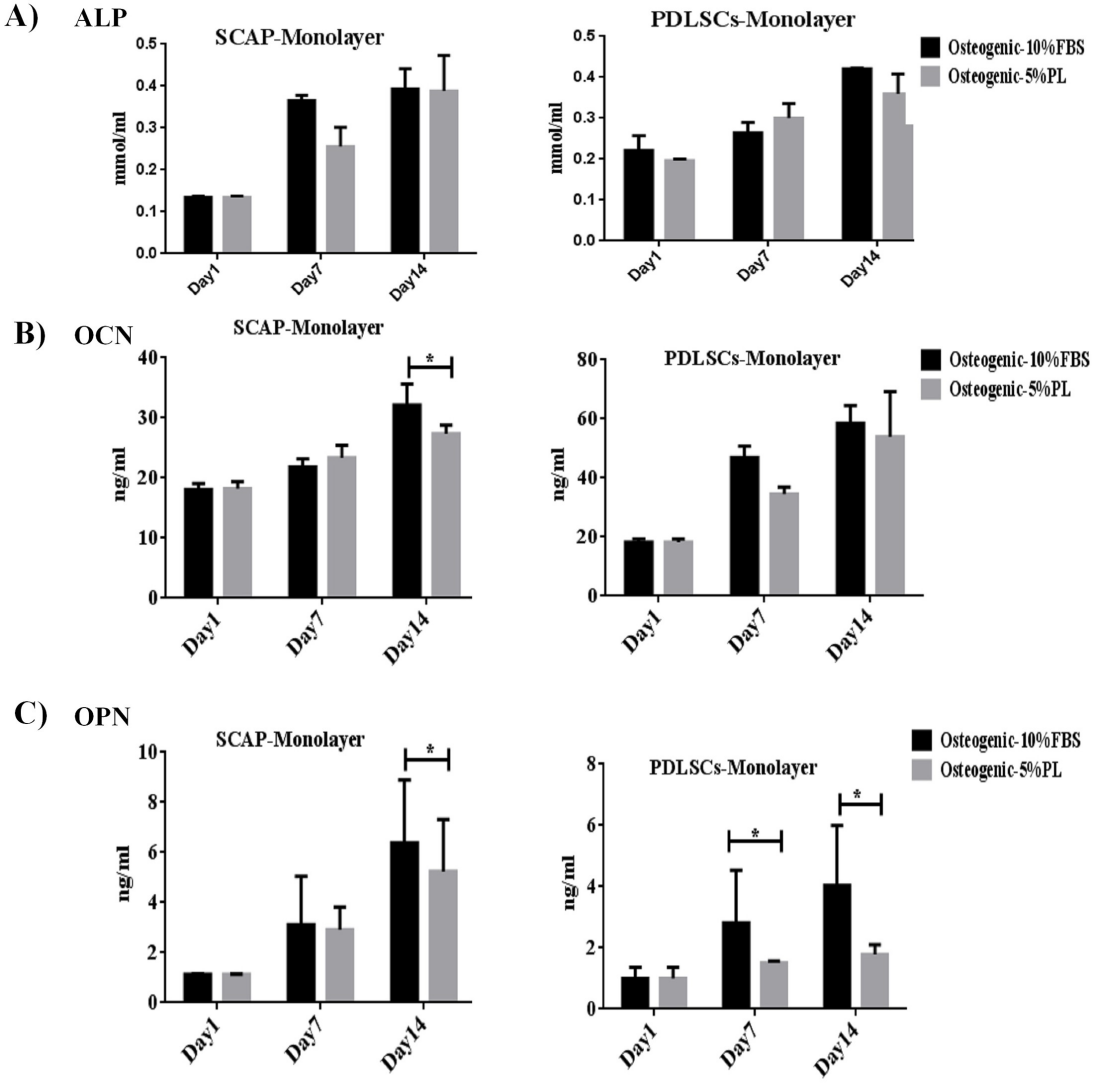
Concentrations of osteogenic markers measured by ELISA of SCAP and PDLSCs cultured as monolayers. Measurement of ALP, OCN and OPN osteogenic markers on monolayer cultures at different time points of osteogenic differentiation (1,7 and 14 days).

On monolayer 2D culture, both serum types were able to significantly increase the secretion of the osteogenic markers (ALP, OCN and OPN) (P<0.05) on both cell types (Fig 3). The expression of ALP increased consistently throughout the differentiation period (Fig 3A). However, the expression of OCN and OPN was higher on 10% FBS culture at day 14 compared to 5% hPL culture for SCAP and PDLSCs (P<0.05) (Fig 3B and 3C) (S3 Dataset).

**Fig 4:**
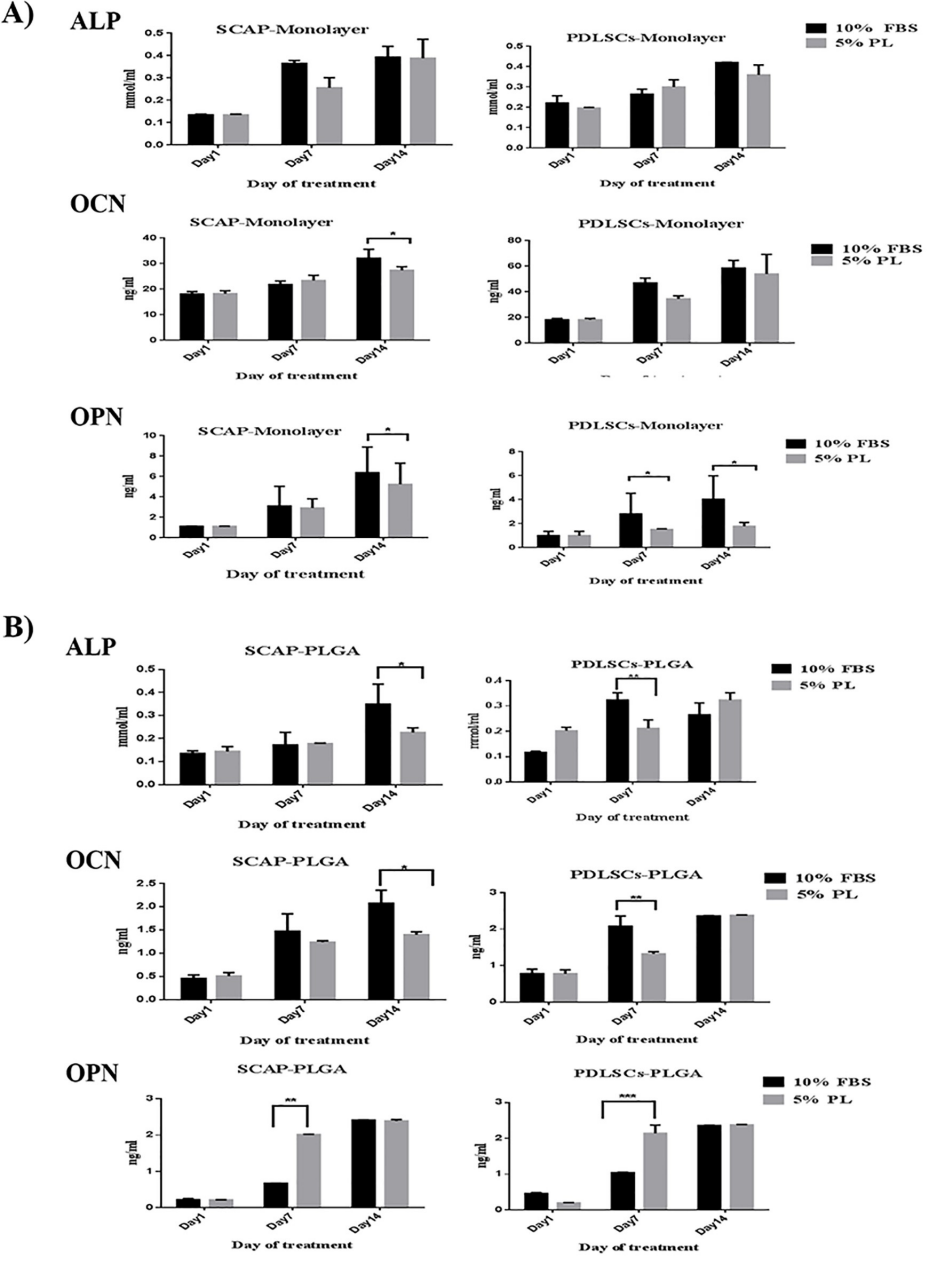
Evaluation of the expression of osteogenic markers measured by ELISA of SCAP and PDLSCs seeded on PLGA scaffold. Measurement of ALP OCN and OPN of cells seeded on PLGA scaffolds (3D) at different time points of osteogenic differentiation (1,7 and 14 days) on media supplemented with either FBS or PL, compared to seeded PLGA cultured in cell culture media without the osteogenic supplements(Blank Control).

For PLGA scaffolds (3D), ALP concentration has been increased steadily throughout the induction period in both cell types under 5% hPL culture conditions (Fig 4A). Whereas, PLGA scaffolds seeded with SCAP and treated with 10% FBS showed a statistically significant difference compared to the scaffolds treated with 5% hPL at day 14 of induction (P<0.05). On the other hand, PLGA scaffolds seeded with PLDSCs and treated with 10% FBS showed a significant increase in ALP level at day 7 of induction compared to cells treated with 5% hPL (P<0.05) (S4 Dataset).

However, PLGA scaffolds seeded with SCAP cells and cultured on 10% FBS culture medium were capable to increase the level of OCN at day 14 compared to scaffolds treated with 5% hPL (P<0.05). Scaffolds seeded with PDLSCs and maintained on 10% FBS culture medium showed statistically significant increase on OCN level at day 7 of induction when compared to 5% hPL culture medium (P<0.05) (Fig 4B).

Interestingly, OPN level on PLGA scaffolds seeded with SCAP or PDLSCs and treated with 5% hPL showed significant increase in the expression level at day 7 as compared to 10% FBS (P<0.05) and the expression remained stable till day 14 (Fig 4C). Overall, PLGA scaffolds (3D) maintained a stable and consistent increase in osteogenic markers expression with 5% hPL throughout the differentiation period when compared to 10% FBS. Interestingly, seeded PLGA scaffolds cultured in osteogenic media showed a significant difference in all of the expression of ALP, OCN and OPN when compared to PLGA scaffolds cultured in cell culture media only (without any osteogenic supplements), regardless the cell type and serum choice (P<0.05) (Fig 4).

## Discussion

In the field of regenerative dentistry, the most motivated aim is to reconstruct a whole tooth along with the surrounding tissues. However, this objective is facing some major challenges such as patient incompatibility, progenitor cells, and the derivation of functional ameloblasts [39, 52]. Therefore, the logical approach is to be able to regenerate a mineralized bioengineered root such as the formation of dentin and cementum which are formed from DPSCs and PDLSCs respectively [12, 47].

Many issues have been raised against the use of MSCs in clinical applications and their expansion in large-scale GMP-compliant protocols [53]. In particular, the use of FBS is complicated due to many reasons; such as high lot-to-lot variability, risk of transmitting infectious agents and immunizing effect. Consequently, hPL has been considered as a possible alternative to FBS for culturing MSCs as it showed a great potential to support MSCs proliferation and differentiation *in vitro* and in maintaining their ability to form bone *in vivo* [40, 47, 53].

DSCs are an attractive source for oral and maxillofacial regenerative therapy. We aimed to assess their ability to give mature and functional osteoblasts when seeded on a polymeric PLGA scaffold (3D) and monolayer culture using 10% FBS and 5% hPL. Our data showed that, both serum types have similar capability to maintain characteristics of the derived DSCs (SCAP and PDLSCs), such as morphological appearance, surface markers analysis, proliferative capacity and multi-lineage differentiation potential.

As observed on H&E and SEM analysis, PLGA scaffolds (3D) were able to enhance the growth and differentiation potential of both cell types. It was clearly shown that cells were able to migrate and penetrate inside the scaffolds by forming thin sheet-like structure and maintaining their osteogenic differentiation capability. Our data are in agreement with previously published data in which the appropriate concentration of hPL enhanced the proliferation and mineralized differentiation of human DPSCs both *in vitro* and *in vivo* [24]. This result supports the use of hPL as an alternative to FBS or a nonzoonotic adjuvant for cell culture. Although DPSCs share some features with PDLSCs and SCAP, they are inherently different, especially with regard to their origin and differentiation ability and therefore their potential clinical relevance in the future. Moreover, ectopic bone formation was observed on ceramics seeded with MSCs grown in hPL medium and implanted under the skin of immunodeficient mice for 7 weeks [54]. In addition to the multilineage differentiation potential of MSCs when cultured on (epsilon-caprolactone) (PCL) scaffold [55].

The functionality of differentiated cells toward osteogenic lineage was examined by measuring the expression levels of ALP, OCN and OPN. ALP was highly expressed at the early stages of osteogenic differentiation, which emphasizes its role in the mineralization process [56]. While, OCN and OPN were highly expressed at late stage of osteogenic differentiation and they regulate the formation of mineral nodules during osteogensis [57, 58]. hPL showed high capacity to give functional osteoblasts by forming mineralized nodules and elevated expression of osteogenic markers such as; ALP, OCN and especially OPN, which increased significantly with time when cells were seeded on PLGA scaffold (3D). Atari et al and his group have reported significant expression of bone markers, calcium deposition and ALP activity during osteogenic differentiation of DPSCs seeded on Cell Carrier glass scaffolds [23]. Similarly, DPSCs seeded on hydroxyapatite-tricalcium phosphate (HA/TCP) biomaterials in culture medium containing 5% hPL significantly promoted the mineralized differentiation of DPSCs, as indicated by the measurement of ALP activity and calcium deposition under mineral-conditioned media [24]. Furthermore, 5% hPL also enhanced mineralized differentiation of DPSCs and SCAP, as indicated by the measurement of ALP activity, OCN, OPN and calcium deposition. Thus, PL is considered as a valid substitute of FBS to culture DPSCs for clinical use [47, 59].

Our work is the first to describe the effect of hPL on promoting osteogenic differentiation of PDLSCs and SCAP seeded in three-dimensional scaffolds. These data are of importance from two different perspectives. First, the scaffold biomaterial used PLGA is widely-studied in the literature both in vitro and in vivo. It is a biocompatible biodegradable product, and its fabrication is easily modified to account for the degree of degradability required. Further, the scaffolds can either be milled into the shape of the defect or they can be fabricated into small "cubes" that would fit into the defect and fill it completely similar to allogenic bone chips. Alternatively, other biomaterials such as HA and TCP, even though they may provide superior osteogenic differentiation of MSCs without the use of osteogenic media, are brittle and cannot be manipulated or "squeezed" into defects. Secondly, most of the data available in literature describe the behavior of cells and regenerative products developed in vitro without consideration of process transfer into clinical applications. Unfortunately, this has hindered the rapid progression of regenerative options in dentistry. Therefore, work such as the one presented here has the advantage of taking this into consideration. Process development of a regenerative product devoid of xenogenic materials and fabricated with proper quality control tests, such as expression levels of ALP, OCN and OPN, should be an easier task than that where all of these factors have to be reconsidered and verified.

## Conclusion

We conclude that hPL could be used as an efficient substitute of xenogenic products to augment osteogenic differentiation of PDLSCs and SCAP when seeded in a three-dimensional scaffold fabricated from the biocompatible, biodegradable PLGA material. This combination of cells/scaffold/growth supplement seems to fulfil an ideal triad required in regenerating periodontal defects.

## Supporting information

S1 Fig FTIR-spectra of the PLGA raw material and the fabricated one. The upper line represents PLGA scaffold and the lower line represents PLGA pellets. Both show similar values.
(TIF)

S2 Fig DSC thermogram of PLGA scaffold (__) and its pellet (---). Both PLGA 3D scaffold and its pellet show similar T_g_ values.
(TIF)

S3 Fig TGA thermogram of PLGA Scaffold (….) and its pellet (). The data show that PLGA scaffold has higher thermal stability than its raw material, the pellet.
(TIF)

S1 Dataset Raw data and statistical analysis of colony forming efficiency (CFE) results of SCAP and PDLSCs cultured in two different serum types, at different passages in addition to their statistical analysis. (XLSX)

S2 Dataset Raw data and statistical analysis of cell proliferation assay (MTT) results for SCAP and PDLSCs cultured in two different serum types for 72 hours. (XLSX)

S3 Dataset Raw data and statistical analysis of osteogenic marker measured by ELISA expressed by SCAP and PDLSCs, when cultured as monolayers at different time points of differentiation. (XLSX)

S4 Dataset Raw data and statistical analysis of osteogenic marker measured by ELISA expressed by SCAP and PDLSCs, when seeded on PLGA at different time points of differentiation. (XLSX)
