# Supplementary figures and images for: hPL promotes osteogenic differentiation of stem cells in 3D scaffolds
Source: PLoS One. 2019 May 7;14(5):e0215667. doi: 10.1371/journal.pone.0215667 (PMC6504042; doi:10.1371/journal.pone.0215667)

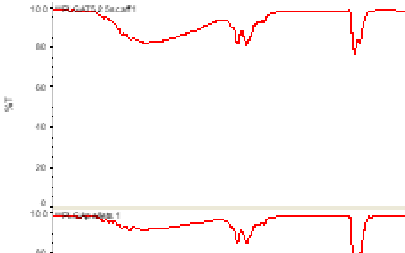

Supplement: S1 Fig — The upper line represents PLGA scaffold and the lower line represents PLGA pellets. Both show similar values. (TIF) [file pone.0215667.s001.tif]

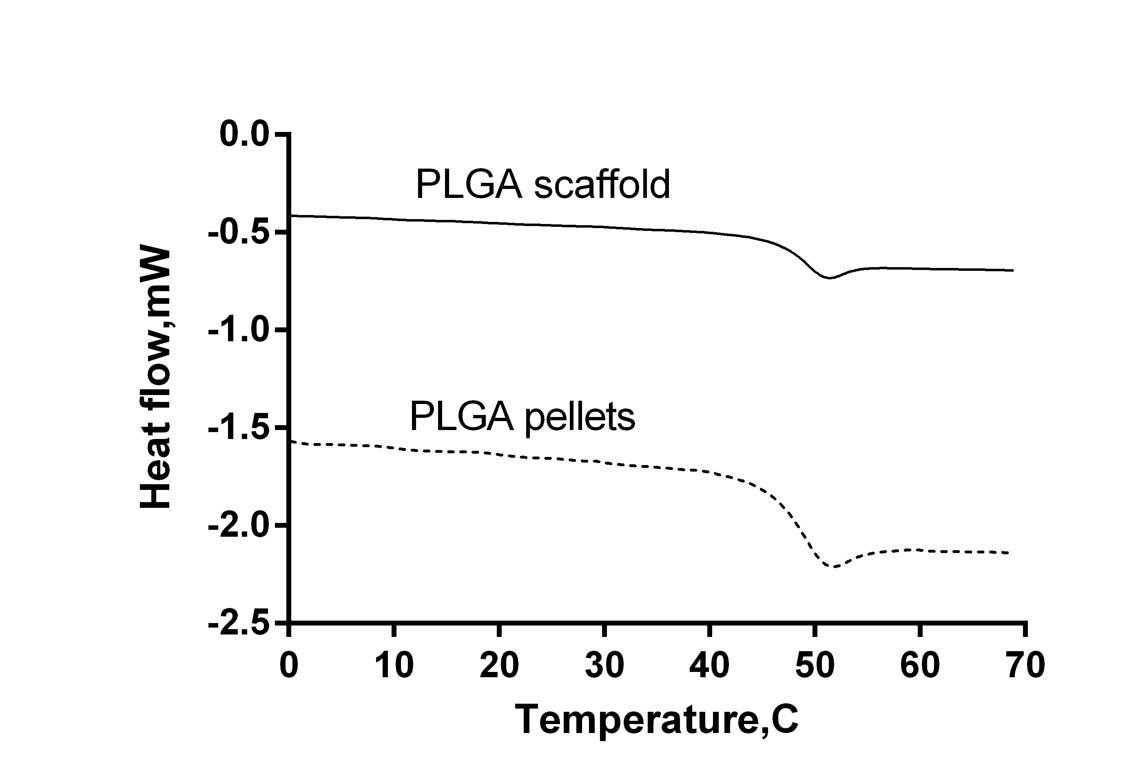

Supplement: S2 Fig — Both PLGA 3D scaffold and its pellet show similar Tg values. (TIF) [file pone.0215667.s002.tif]

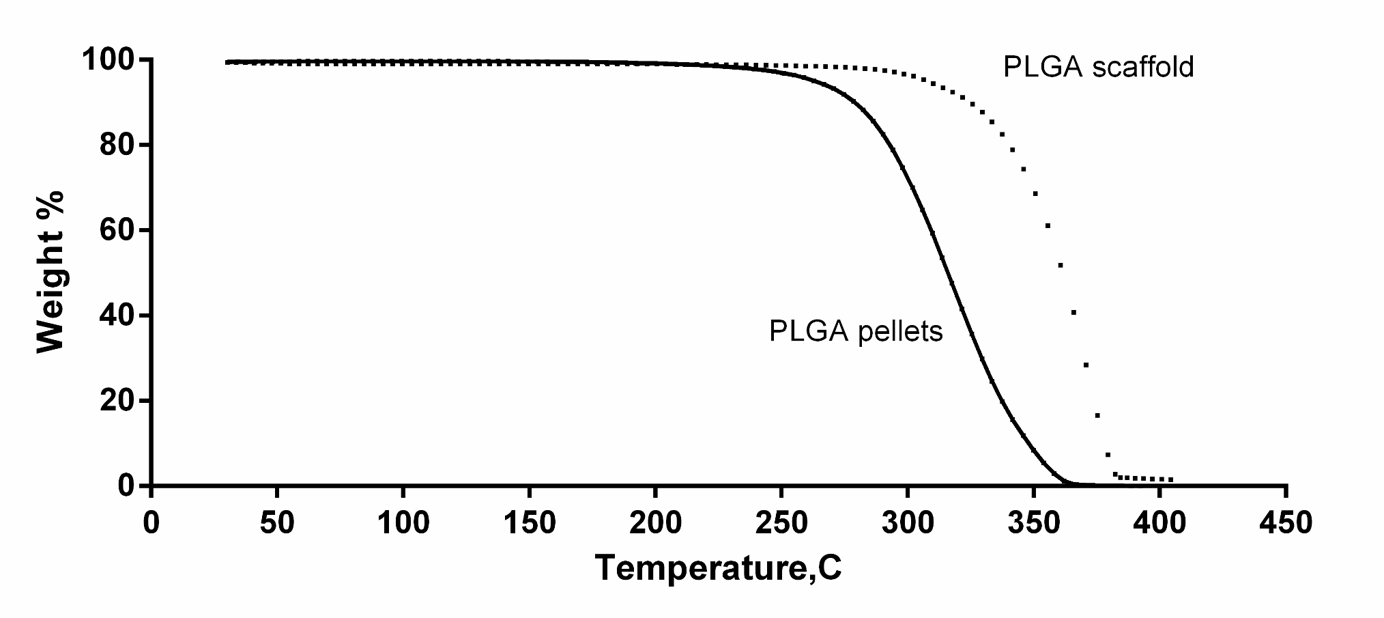

Supplement: S3 Fig — The data show that PLGA scaffold has higher thermal stability than its raw material, the pellet. (TIF) [file pone.0215667.s003.tif]
